# Supplementary figures and images for: Multiple light signaling pathways control solar tracking in sunflowers
Source: PLoS Biol. 2023 Oct 31;21(10):e3002344. doi: 10.1371/journal.pbio.3002344 (PMC10617704; doi:10.1371/journal.pbio.3002344)

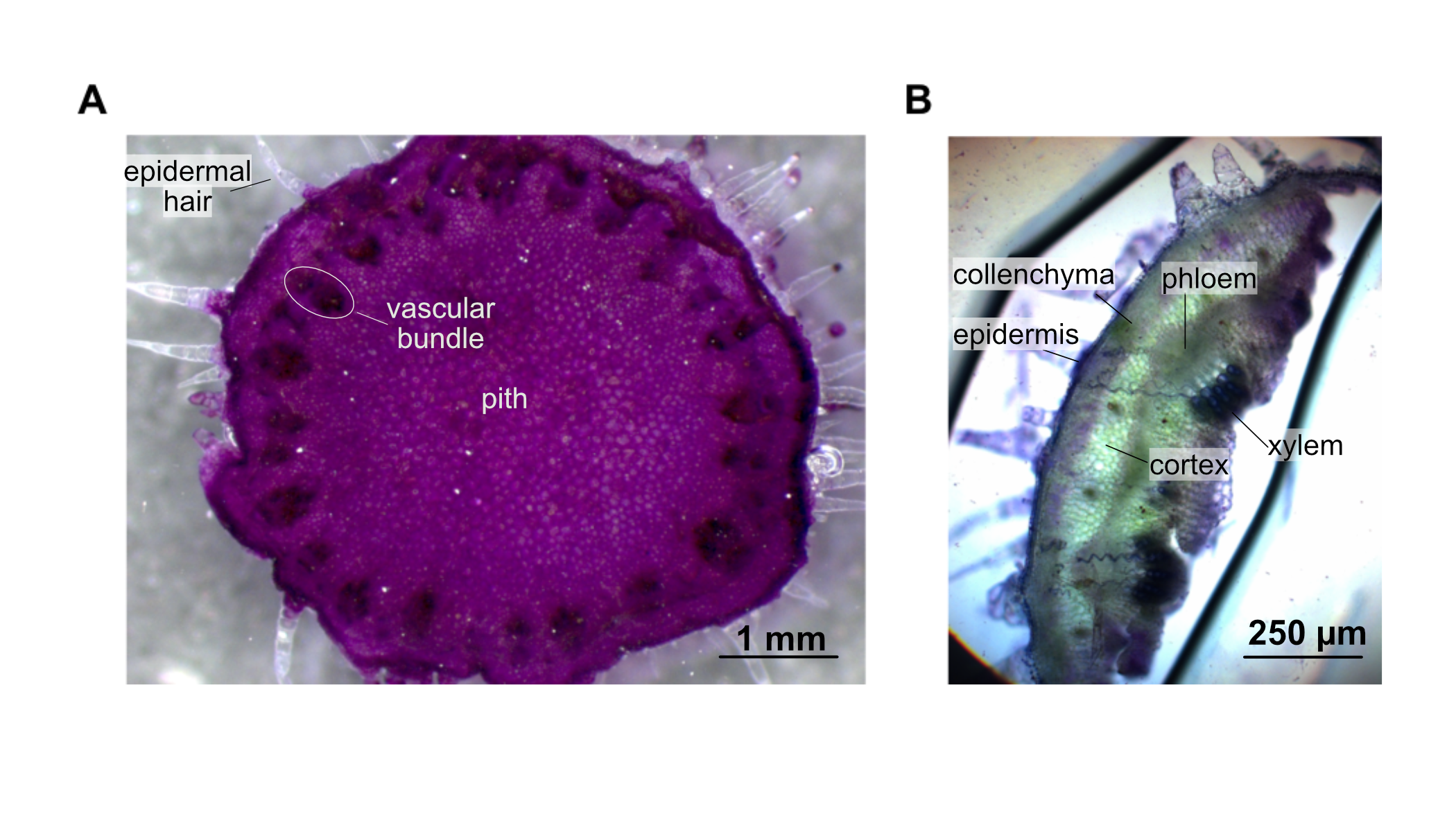

Supplement: S1 Fig — (A) Cross-section of whole sunflower stem. Stem section stained with 0.05% toluidine blue and image taken at 25× magnification using a dissection scope. (B) Cross-section of stem peel stained with 0.01% toluidine blue and image taken at 100× magnification using a Zeiss Axioskop 2 plus. (TIFF) [file pbio.3002344.s001.tiff]

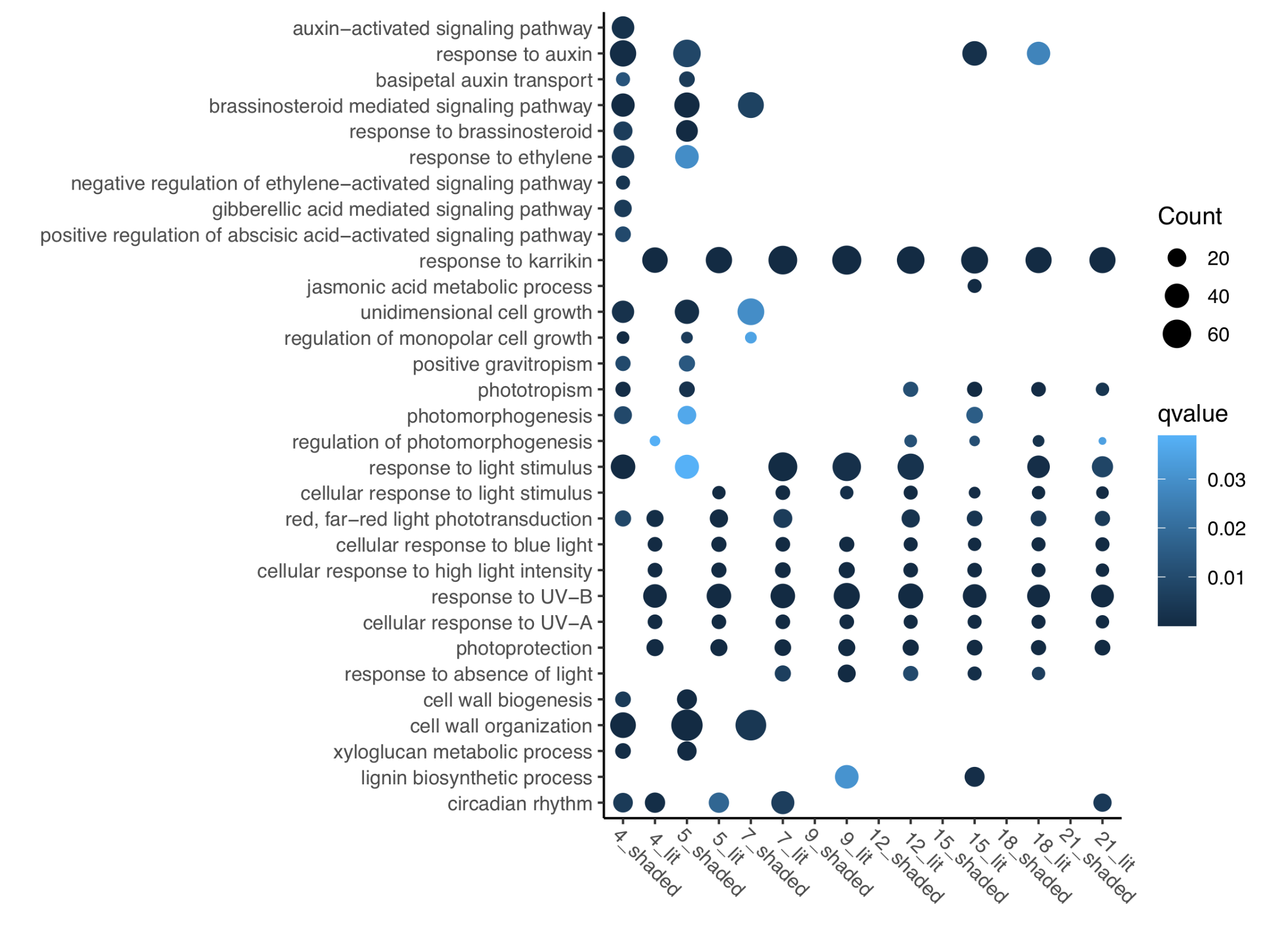

Supplement: S2 Fig — GO enrichment analysis of genes differentially expressed genes during phototropism between ZT4 and ZT21. All terms for processes involved in light signaling, hormone regulation, and growth are shown for lit and shaded sides of stems. The underlying raw data may be found in S2 Data. (TIFF) [file pbio.3002344.s002.tiff]

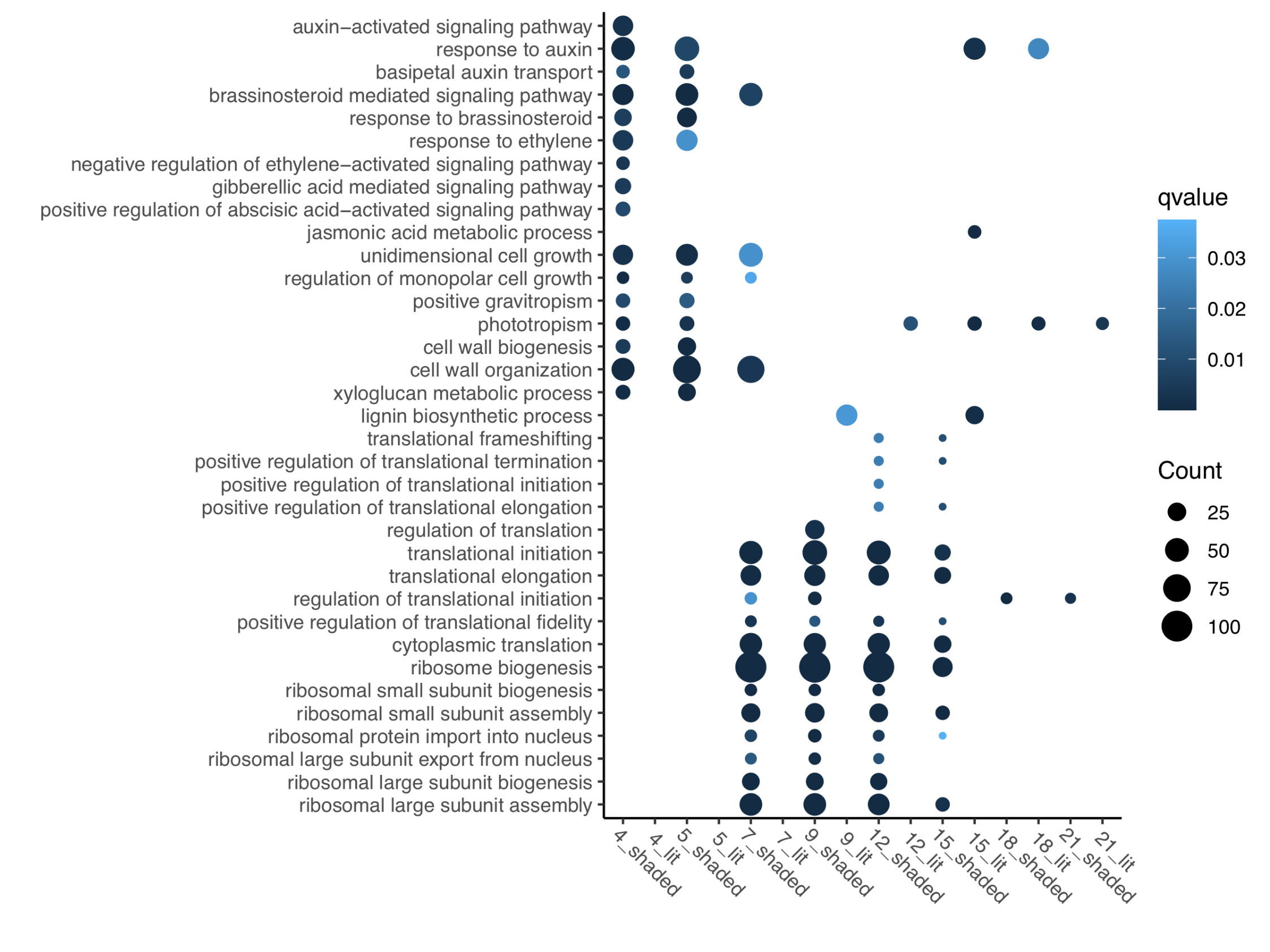

Supplement: S3 Fig — GO enrichment analysis of genes differentially expressed genes during phototropism between ZT4 and ZT21. All terms for processes involved hormone regulation, growth, cell wall–related processes, translation, and ribosomal processes are shown for lit and shaded sides of stems. The underlying raw data may be found in S2 Data. (TIFF) [file pbio.3002344.s003.tiff]

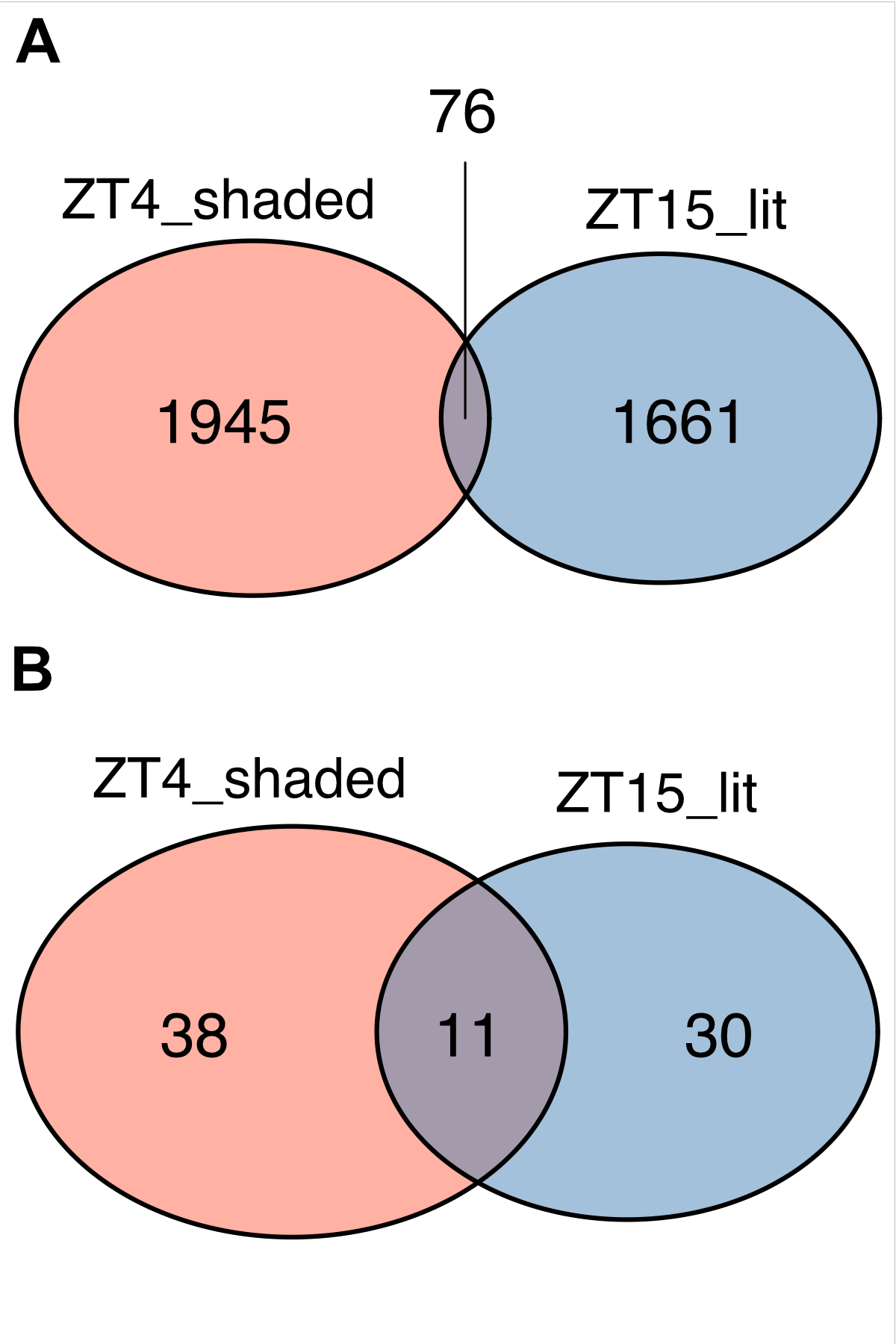

Supplement: S4 Fig — (A) Venn diagram of all up-regulated genes on the shaded side at ZT4 and lit side at ZT15 in phototropic plants. (B) Venn diagram of genes assigned the “Response to Auxin” GO term that are up-regulated on the shaded sides of stems at ZT4 and on the lit sides of stems at ZT15 in phototropic plants. The underlying raw data may be found in S3 Data. (TIFF) [file pbio.3002344.s004.tiff]

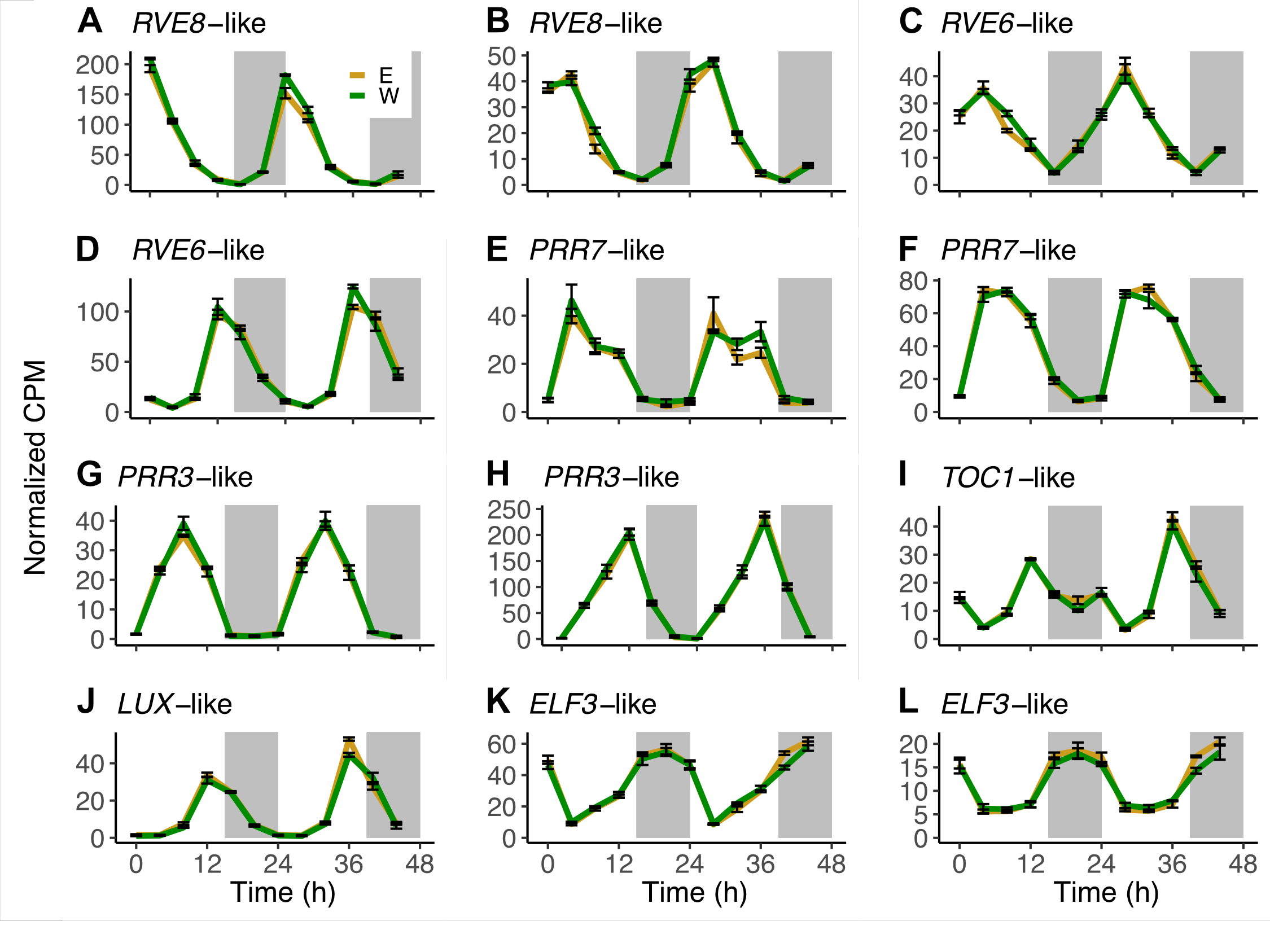

Supplement: S5 Fig — (A) RVE8-like (Ha412HOChr07g0311811). (B) RVE8-like (Ha412HOChr14g0679651). (C) RVE6-like (Ha412HOChr16g0792081). (D) RVE6-like (Ha412HOChr09g0395701). (E) PRR7-like (Ha412HOChr08g0341571). (F) PRR7-like (Ha412HOChr01g0042171). (G) PRR3-like (Ha412HOChr14g0684881). (H) PRR3-like (Ha412HOChr07g0319851). (I) TOC1-like (Ha412HOChr02g0065721). (J) LUX-like (Ha412HOChr08g0371471). (K) ELF3-like (Ha412HOChr07g0293611). (L) ELF3-like (Ha412HOChr02g0074131). (A-L) Normalized expression over time for stems undergoing heliotropism (mean +/− SEM, n = 3). The underlying raw data may be found at NCBI GEO, accession GSE229654. (TIFF) [file pbio.3002344.s005.tiff]

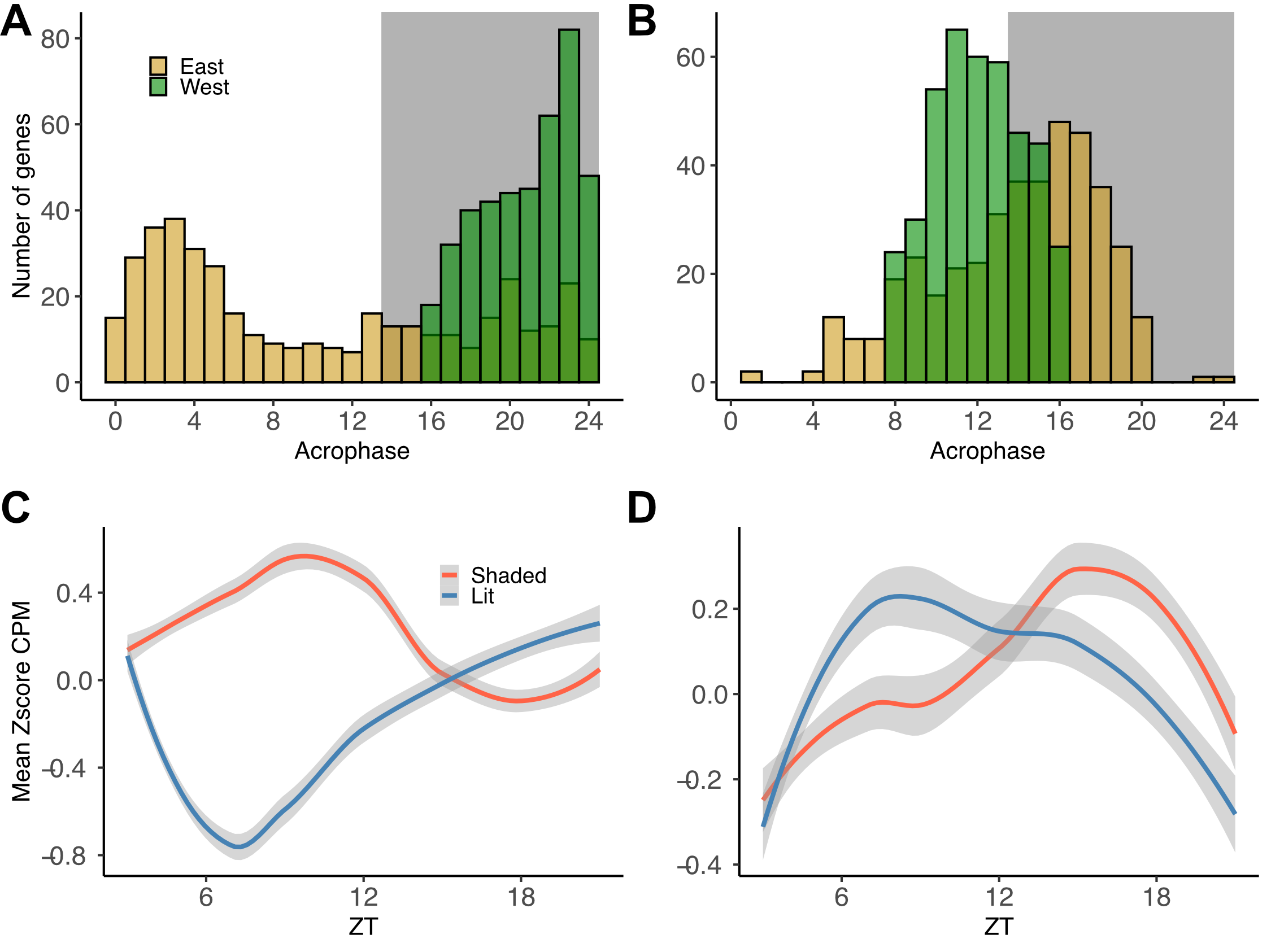

Supplement: S6 Fig — (A, B) Genes with different acrophases on the east and west sides of stems; west side acrophases between ZT16 and ZT24 (A) or between ZT8 and ZT16 (B). (C, D) Zscore-normalized mean expression of these differentially phased genes (shown in A, B) during phototropism. Lines fitted with LOESS. Ribbons represent 95% confidence intervals. The underlying raw data may be found in S6 Data for S6A and S6B Fig and in S17 Data for S6C and S6D Fig. (TIFF) [file pbio.3002344.s006.tiff]

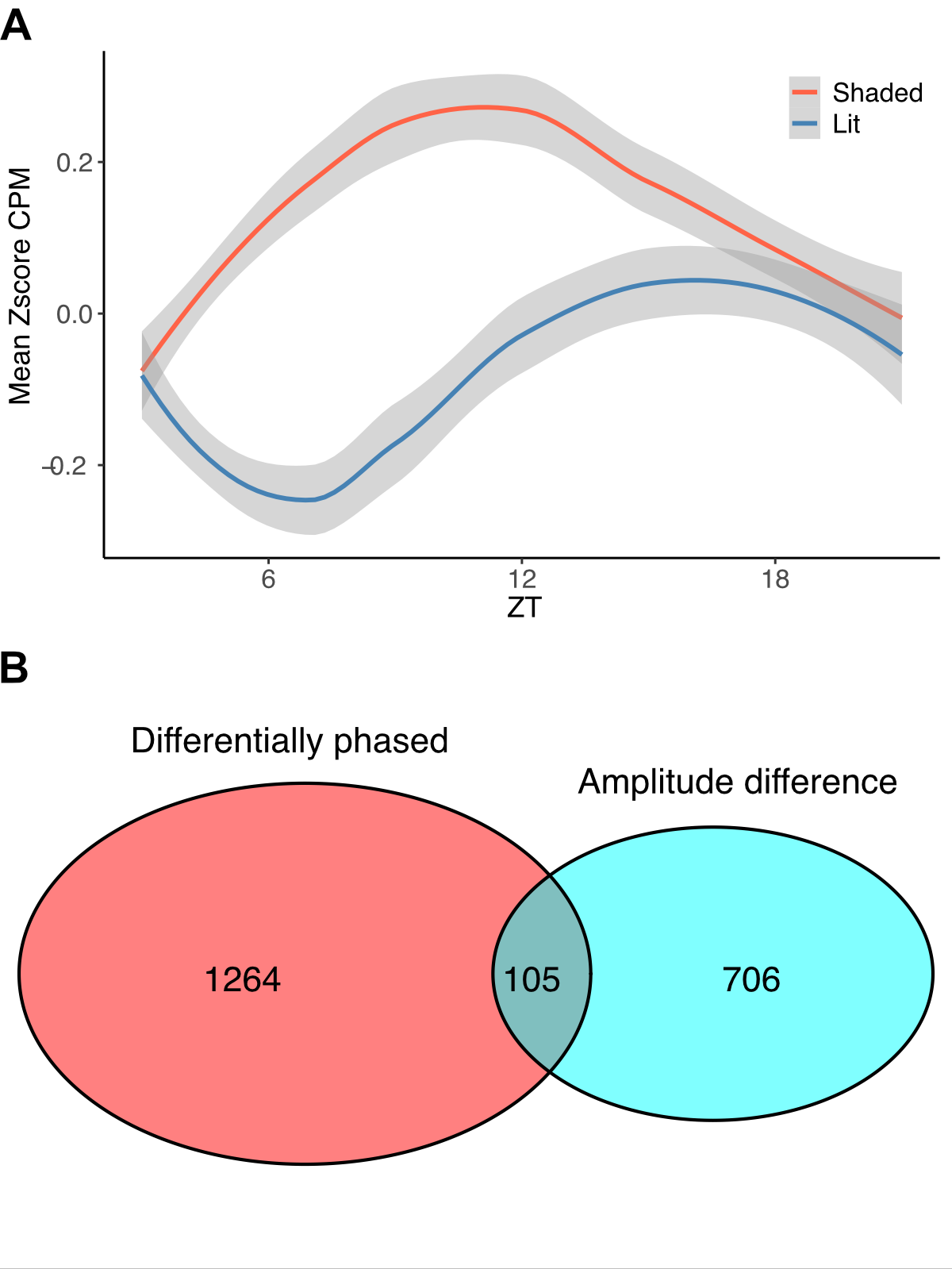

Supplement: S7 Fig — (A) Genes with a >2-fold difference in amplitude between east and west sides of stems during heliotropism. Zscore-normalized mean CPM of these genes during phototropism. Lines fitted with LOESS. Ribbons represent 95% confidence intervals. (B) Venn diagram showing overlap between those genes with an amplitude difference during heliotropism and genes with a phase difference during heliotropism. The underlying raw data may be found at NCBI GEO, accession GSE229654 and in S7 and S17 Data for S7A Fig; and in S6 and S7 Data for S7B Fig. (TIFF) [file pbio.3002344.s007.tiff]

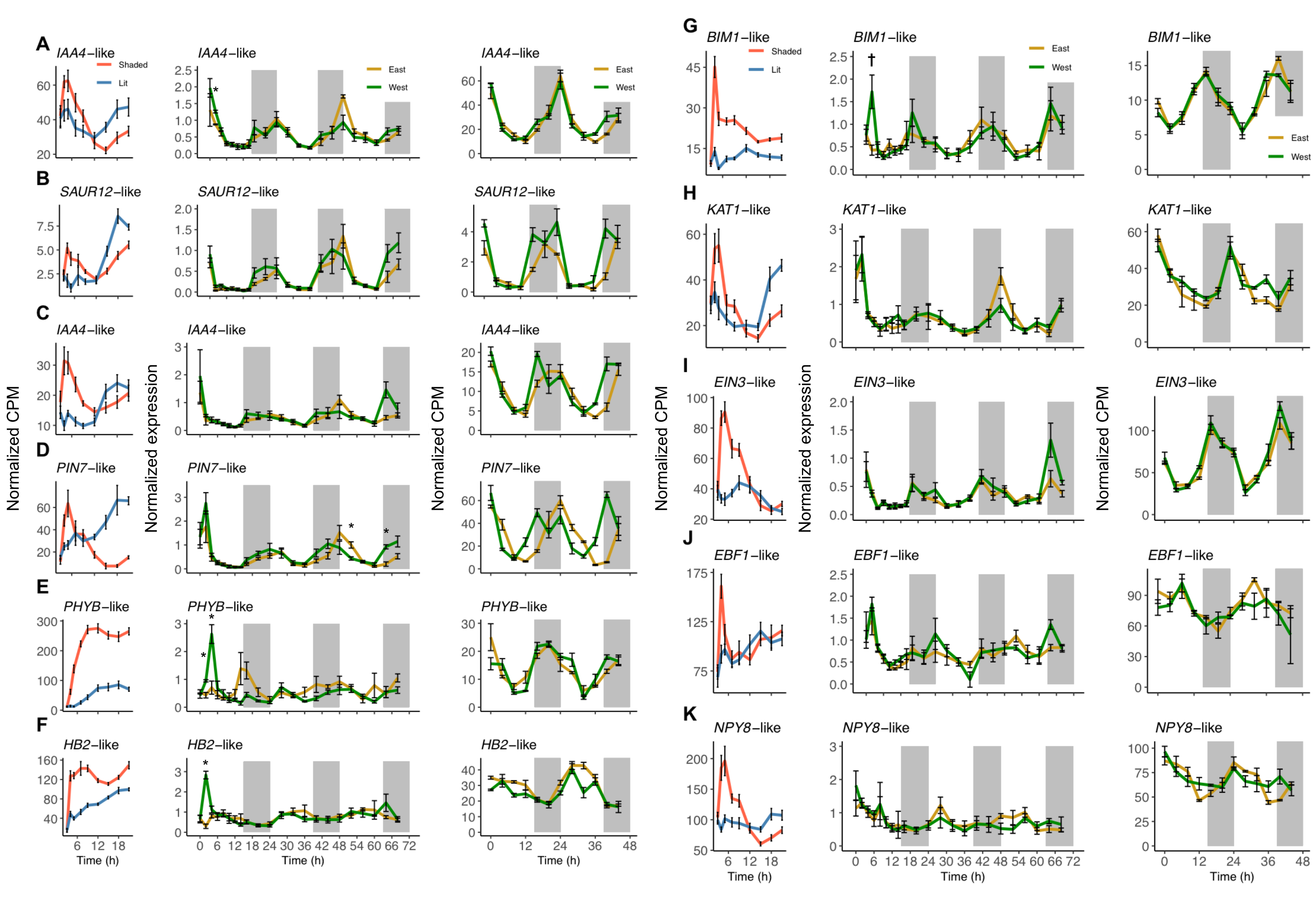

Supplement: S8 Fig — (A) IAA4-like (Ha412HOChr10g0435441). (B) SAUR12-like (Ha412HOChr07g0303111). (C) IAA4-like (Ha412HOChr01g0004341). (D) PIN7-like (Ha412HOChr01g0035481). (E) PHYB-like (Ha412HOChr02g0079361). (F) HB2-like (Ha412HOChr13g0623611). (G) BIM1-like (Ha412HOChr02g0064141). (H) KAT1-like (Ha412HOChr04g0179931). (I) EIN3-like (Ha412HOChr15g0726161). (J) EBF1-like (Ha412HOChr01g0047431). (K) NPY8-like (Ha412HOChr12g0580251). The left graph in all panels is the normalized expression over time on shaded and lit sides of stems (mean +/− SEM, n = 6) undergoing phototropism in a growth chamber. The middle graph for all panels is normalized expression during the first 3 days in the field (mean +/− SEM, n = 3, except ZT64 west, n = 2). Statistical significance calculated using Welch’s t test, qvalue < 0.05. The right graph for each panel is normalized expression over time for stems maintaining heliotropic movements (mean +/− SEM, n = 3). The underlying raw data may be found in S14 Data for the middle plots, and at NCBI GEO, accession GSE229654, for the plots on left and right sides of the figure. (TIFF) [file pbio.3002344.s008.tiff]

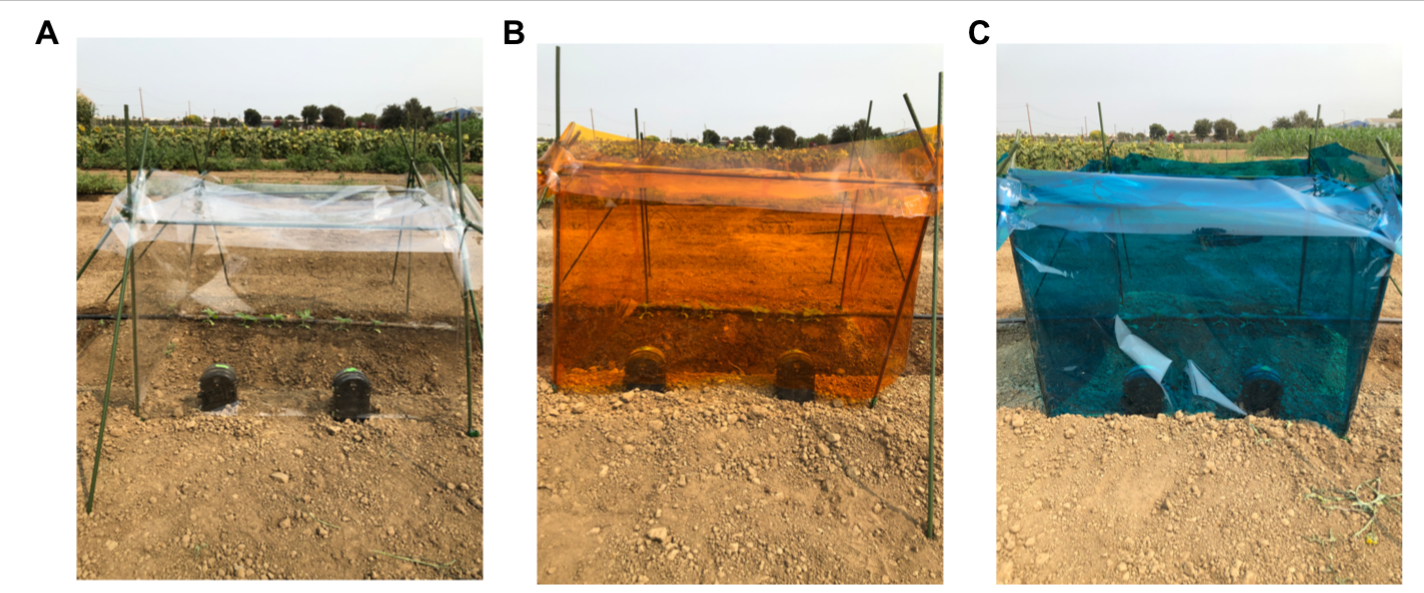

Supplement: S9 Fig — Photos taken in the field of light filter boxes, (A) clear box, (B) blue-depleting box, (C) red-depleting box. (TIFF) [file pbio.3002344.s009.tiff]

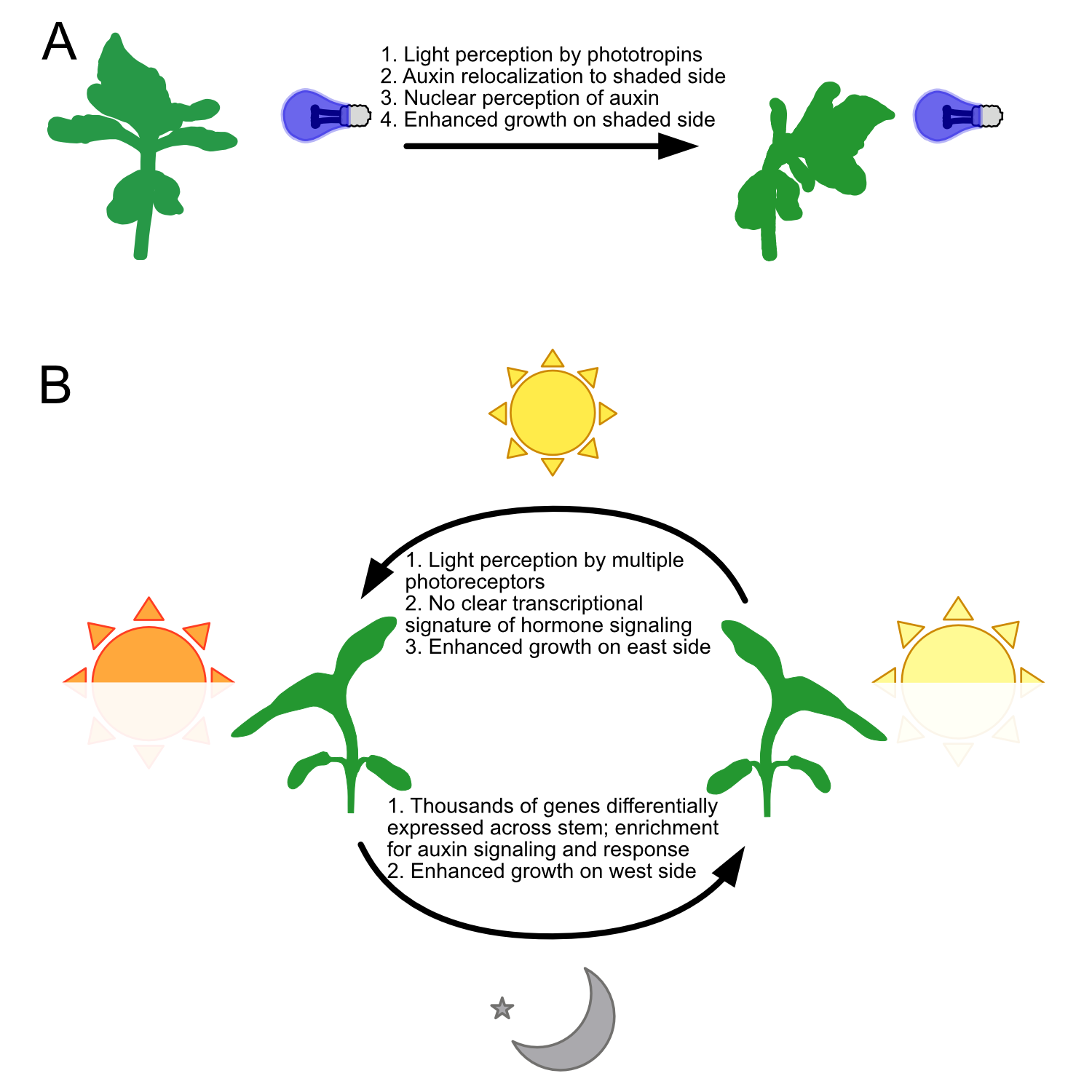

Supplement: S10 Fig — (A) Sunflower plants bending towards blue light in a growth chamber show transcriptional responses typical of the phototropin signaling pathway. (B) Daytime heliotropic movements likely depend on multiple types of photoreceptors but do not display transcriptional patterns associated with specific hormone signaling pathways. Nighttime heliotropic movements are associated with extensive differential gene expression across the stem, with enrichment for auxin signaling and response genes on the faster growing, west sides of stems. (TIFF) [file pbio.3002344.s010.tiff]

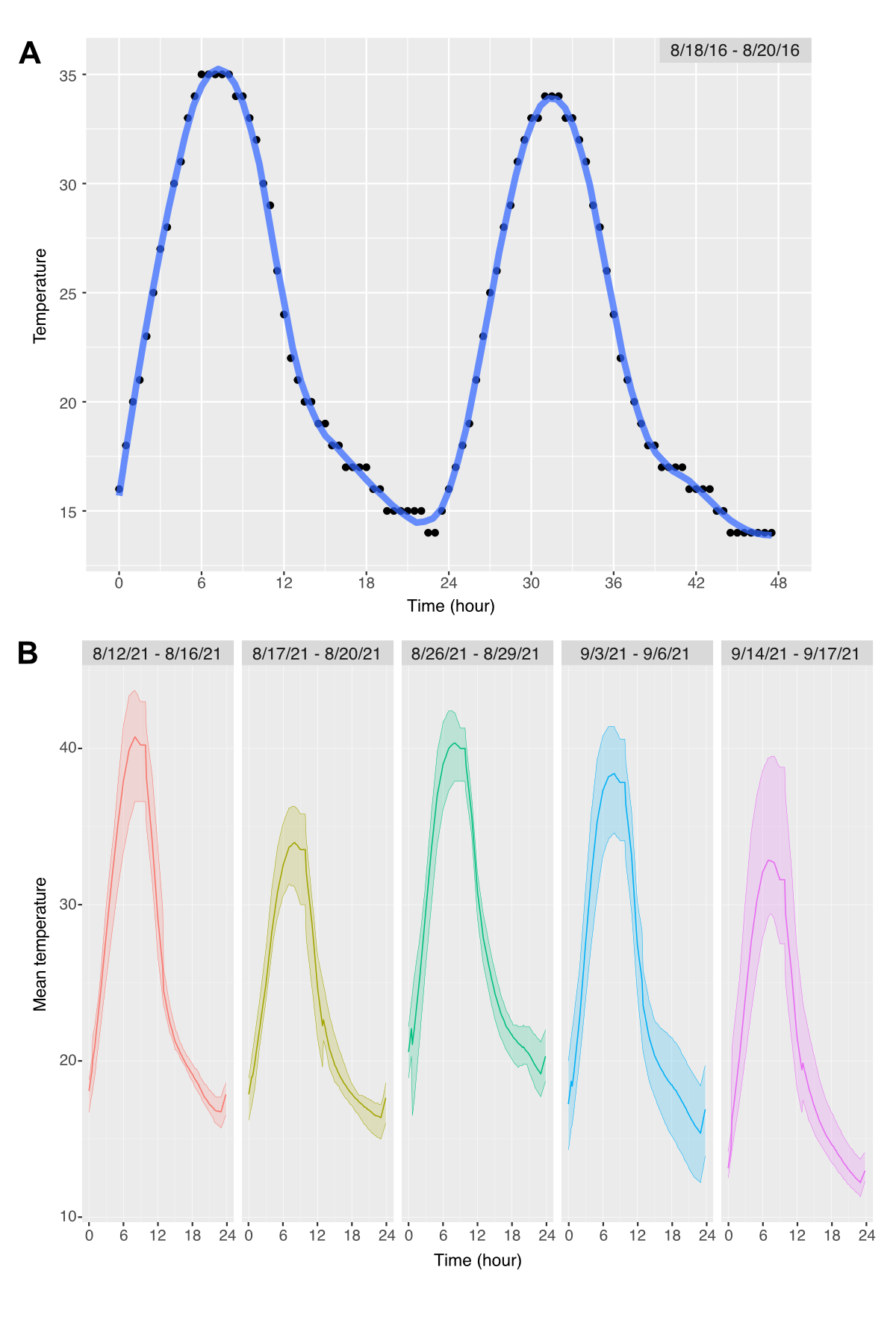

Supplement: S11 Fig — (A) Temperatures during collection of tissue samples for RNA-seq analysis of gene expression during heliotropism. (B) Average temperatures during each filter box trial. Ribbons represent the minimum and maximum temperatures during each trial. All temperatures are in degrees Celsius. The underlying raw data may be found in S16 Data. (TIFF) [file pbio.3002344.s011.tiff]

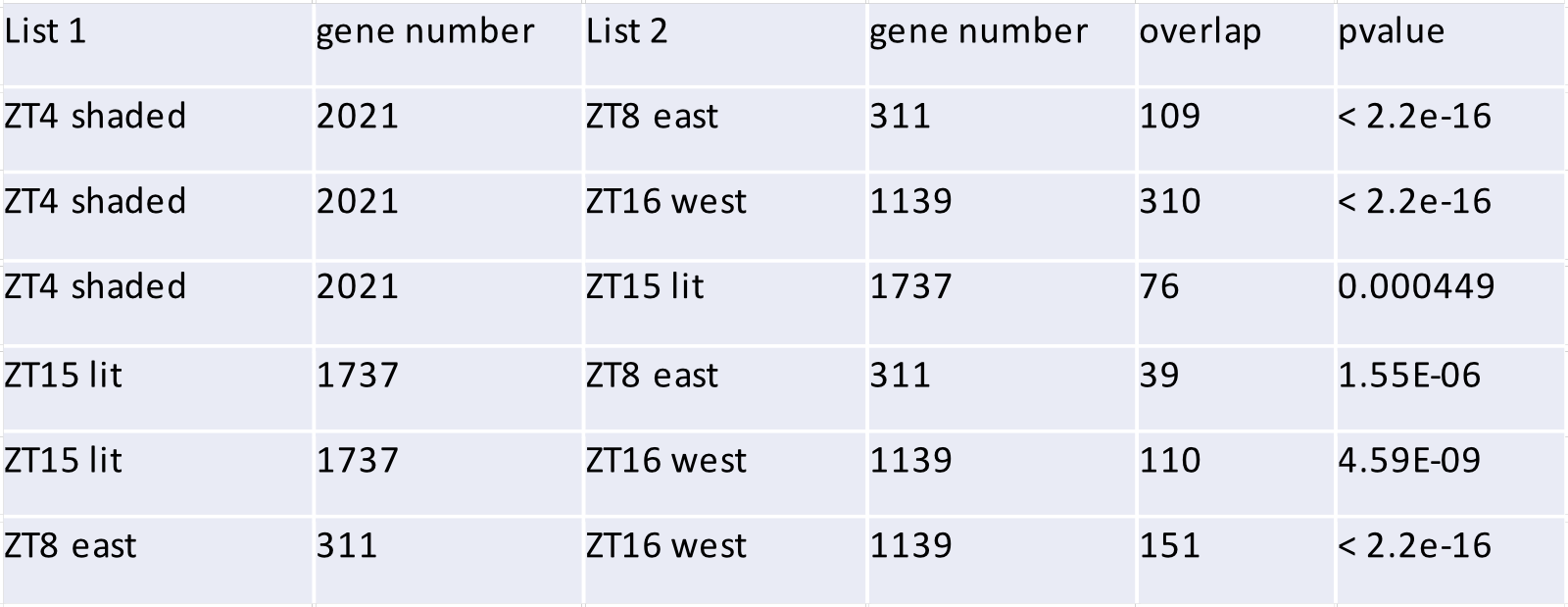

Supplement: S1 Table — Fisher’s exact test run on each combination of the overlap of differentially expressed genes as shown in Fig 3M. (TIFF) [file pbio.3002344.s012.tiff]
