# Supplementary material for: Multiple light signaling pathways control solar tracking in sunflowers
Source: PLoS Biol. 2023 Oct 31;21(10):e3002344. doi: 10.1371/journal.pbio.3002344 (PMC10617704; doi:10.1371/journal.pbio.3002344)
Supplement: Attachment — Submitted filename: Response_to_reviewers_PLoS_Bio.docx [file pbio.3002344.s031.docx]

**Response to reviewers**

Dear Dr. Alvarez-Garcia,

Thank you for the opportunity to submit a revised version of our manuscript “Transcriptome and physiological analyses implicate multiple light signaling pathways in the control of solar tracking in sunflower”. We are pleased that you and the reviewers found the work interesting and appreciate the constructive suggestions on how to improve it. We have incorporated the reviewers’ comments into this revised version, as detailed below. We hope that you now find it suitable for publication in *PLoS Biology*.

Sincerely,

Stacey Harmer

**Reviewer’s comments**

**Reviewer #1**

In this manuscript by Brooks et al., the authors study how sunflower plants track the sun as it moves across the sky each day, a process known as heliotropism. Previously, it was predicted that sunflower heliotropism is a specialized form of phototropism, the bending of plants towards light, but this has not been shown empirically. They use two main techniques to address this question, physiological measurements of stem bending and transcriptomics. They grow sunflower plants in a phototropic light regime in which blue light is held in place to trigger phototropic response. Then they grow plants in heliotropic conditions, including outdoor grown sunflower exhibiting heliotropic movements and indoor grown sunflower plants not exhibiting heliotropic movements but then transferred outdoors where they begin heliotropic movement. They measure stem angles under each of these conditions and collect timecourse RNA-seq samples from stem sections that are either "shaded" (pointed away from the light source) or "lit" (pointed toward a light source). They then use the RNA-seq to track transcriptional changes and compare these changes between plants undergoing phototropism to plants undergoing heliotropism. From these experiments, they find that heliotropism and phototropism show different transcriptional changes. Most prominently, they show that changes in genes responsive to auxin are a core part of phototropic responses but are not a prominent part of heliotropism during the day, only at night. Instead, heliotropism shows changes in genes related to shade avoidance. This prompted them to grow sunflower plants outdoors in heliotropic conditions with either blue or red light filtered out of their environment, and they found that heliotropism continued under either of these conditions. From this work they conclude that heliotropism is not simply a specialized form of phototropism, but rather it constitutes a different type of directional light response that relies on a combination of light signaling pathways.

This work addresses an important, unanswered, and technically challenging question in the field of plant rhythms, what cellular systems do plants use to track the sun as it moves across the sky? Because most model species do not have obvious heliotropic movements, the authors use sunflower plants, which have heliotropic movement but lack genetic tools. Thus, they use transcriptomics and generate an impressive dataset to establish some fundamental ideas about heliotropic movement. I have no major concerns with the approach. I do have a few concerns about the analyses and whether there are additional comparisons that might reveal more about the nature of heliotropism. I also have some suggested ways to improve the clarity of the manuscript. These are outlined below.

We are grateful for your general enthusiasm for the manuscript.

1. I had a bit of trouble following the logic of the paper since there were many dimensions of data that were being explained (i.e. time, stem illuminance, growth condition). I wonder if there is a way to create some simple diagrams or models that can be included in the main figures that make the experimental design more readily accessible to the reader? At the end one could create a summary model that incorporates the results with the diagrams demonstrating the larger conceptual idea.

Thank you for this helpful comment. We have added new panels to figures 1 and 2 to schematically demonstrate the results in these figures were obtained from experiments carried out in growth chambers and in the field, respectively. In figures 3 and 4, we have added small icons (light bulbs and suns) to each panel to indicate whether the experiment was performed in growth chambers or the field. Finally, we have added a new figure (Supplemental Figure 10) to summarize our conclusions about the mechanisms underlying phototropism compared to heliotropism.

2. I think overall there could be increased clarity in the written presentation of the data. This might include clearer justifications for the experiments at the beginning of each section. For instance, I had a bit of trouble understanding why the autostraightening was important to the manuscript. This is likely my own lack of knowledge, but there could be bit more conceptual justification. My thought is that autostraightening could potentially explain some of the heliotropic effect, but that was not clearly stated. I think increased justification and explanation would help with each of the large experiments presented in the manuscript.

This was also very helpful. We have more explicitly explained our motivations at many points in the manuscript (115 – 119; 155 – 156; 211; 282 – 283; 322 – 323; 379 – 381; 437 – 439). We hope that this improves the clarity of the manuscript.

3. Very large expression datasets from plants collected at multiple timepoints were generated here, but the data seem to be underutilized in the comparative analyses. It was unclear why only certain timepoints were chosen for comparisons. For instance, only ZT4 and ZT15 were chosen for the phototropism data when compared to the heliotropic data. Why wasn't there a more comprehensive comparison of the heliotropic data to more timepoints in the phototropic data? This may have revealed that other timepoints in the phototropic data matched the heliotropic data. In this sense, it seems like a lot of the data that was collected wasn't discussed in detail.

We did compare the results of the entire phototropism dataset to those of the heliotropism dataset, but found that timepoints ZT4 and ZT15 accurately encapsulated the changes in changes in gene expression on the shaded and lit sides of the stems. We have added a new figure (Supplemental Fig S2) displaying significantly enrichment GO terms related to hormone signaling and cell growth (the same terms used to generate figures Figs 1G and 2M) for all time points. ZT4 on the shaded sides of the stem contains all the enriched terms found on that side at later time points. ZT15 on the lit sides of stems contains all the enriched terms found on the lit sides of stem; in addition, this the first time point when the response to auxin term is significant.

4. It was unclear why the ZT15 timepoint was chosen as the autostraightening point when comparing to heliotropism. From the phototropic stem bending figure, that seemed like a timepoint that would be too late to catch the initial gene expression changes that would have led to autostraightening. Please explain this in more detail, and if necessary, compare to other autostraightening timepoints.

In retrospect, a time between ZT12 and ZT15 probably would have been ideal for investigating the initiation of the autostraightening response. However, our current analysis does reveal the interesting finding that the ‘response to auxin’ GO category is significantly enriched on the lit sides of autostraightening stems (see response to point 3, above). And as noted above (response to point #3), ZT15 is the first time point with significant enrichment of this auxin-related term (Supplemental Figure S2).

**Reviewer #2**

In their manuscript the authors address the molecular basis of heliotropism, and provide with evidence that this response is different from phototropism, as genes rapidly responsive to phototropism, present only minor changes during heliotropism. Importantly data supports the involvement of not only blue light receptors, but potentially the Red/FR light phytochromes in solar tracking, showing that heliotropism is not a specialized of phototropism dependent on phot-blue light receptors. This is an important basepoint to dissect the complexity of differential growth responses where circadian and light signals interact.

The transcriptomic study conducted is extensive, and most conclusions supported by the study.

Thank you for your positive comments.

However, some comments and suggested adjustments follow.

Material and Methods.

1. Figure S1 illustrates the cross sections of the stems and stem peels used. The figure itself needs indication of the main structures shown. It is also not clear in the material and methods, how the use of the peels led to the dissection of the west and east samples used in the study. How did the dissection happened?, what was the criteria at the cellular level to decide on sampling and ensure samples here as homogeneous as possible? was a criteria of cell size applied in addition to location of the cell? or?

Thank you for this comment. We have added labels to Supplemental Fig S1 to illustrate the main structures of the stem in our peels. We have also modified the Materials and Methods section to clarify that the peels were not microdissected; instead, RNA was extracted from intact peels (lines 692 - 696).

2. Can the authors include in the filter box assays the other environmental factors that impact on growth responses (i.e temperature, PAR differences, photoperiodic light/dark variation).

We have added additional information about the light conditions within each box and during each trial (see lines 757 – 759 and 767 – 769). We have also generated a new figure, Supplemental Fig 11B, showing the mean, minimum, and maximum temperatures during each filter box field trial.

Results/Discussion

1. Figure 1D. In their analysis of phototropism and genes upregulated in the shaded side of stems, they mention Auxin genes were as expected prevalent, but also genes involved in GA and BR sensing/signaling. BZR1 is in Fig 1D provided as example, but can the authors provide with a table of the rest of the hormonal pathways related genes ? Same for the auto-straightening response. How many and which gene homologues of auxin genes were identified? (Fig 1E, 1F).

The supplemental data files listing the statistically significantly enriched GO terms (Data S2, S5, S9, and S10) have a column entitled “geneID” that lists the accession numbers of all the sunflower genes assigned to each GO category. This, along with the new annotation we have provided (Data S11), will allow interested readers to see the sunflower genes and their Arabidopsis homologs present in all the enriched GO categories. We have pointed out the sunflower gene accession numbers are available in these files on lines 178 – 179.

2. Figure 2D, 2E and S1 data. The authors mention in the text that homologues of clock genes displayed strong daily rhythms, and provide the profiles for LHY and PRR5. Probably it would be of interest to include the rest of the clock genes singled out in the analysis ( as part of the S1 data or in a linked table?).

We have generated a new supplementary figure (Supplemental Fig S5) showing the mean expression profiles of 12 homologs of Arabidopsis clock genes on the east and west sides of solar tracking stems. We have also uploaded a file to NCBI GEO (accession # GSE229654) with the normalized counts for all the libraries we generated. This, in combination with the annotation file (Supplemental Data S11) will allow interested readers to examine the expression profiles of all sunflower genes homologous to all Arabidopsis genes of interest.

3. Figure S2 Can the authors provide in Supplementary Tables the gene list related to this figure?

We have generated a new supplemental file (Supplemental Data S3) that provides the identifiers for the sunflower genes in each individual category and those that are in overlapping categories. This file also provides the accession numbers for the closest Arabidopsis homologs of these genes.

4. For the field-experiment with grown sun flower. and expression of genes in the east and west side of the stem. Can the authors comment on the other environmental conditions that applied to the experiment (i.e temperature, dark/light hours, light quality spectra) PAR, R:FR ratio.

Thank you for pointing out this omission. We have added information about the daylength during this trial (lines 696 – 698) and added a figure showing hourly temperatures during this period (Supplemental Figure S11A).

5. For the same experiment, While the authors describe some of the enriched functions of the genes identified in the afternoon and evening (ZT8-12) (ZT12-16),some of the enriched processes like cell division (73 genes) and RNA modifications (ZT12-16- 145 genes) as well as BR signaling (46 in ZT20-24) or Defense genes (83), root development were also enriched. While the last two categories may not be directly linked, can the authors include a comment on the other enriched ones that may have a link or lead to novel unexplored areas?

We have added some speculation regarding possible time-of-day-specific functions for genes implicated in RNA modifications (lines 265 – 268), brassinosteroid signaling (lines 268 – 272), and defense signaling (lines 272 – 274).

6. In the transcriptomic analysis of auxin signaling in heliotropic growth. Can the authors include in supplementary tables the 1369 genes that have acrophase difference greater than 3 hours , those that have different wave forms on the two sides (Fig 2J) ad the 811 with two fold difference in amplitude across the stem?

We have included two new supplemental data files with this information. Supplemental Data 6 lists all genes with a greater than 3-hour difference in acrophase on the east and west sides of stems. Supplemental Data 7 lists genes with a greater than two-fold difference in amplitude across the stem. The gene identifiers of the Arabidopsis homologs of these genes are also included in these files.

7. The authors mention that in their solar tracking experiment, they found enrichment of blue light and UV-responses on the east sides of stems in the morning and evening. A list of the genes identified would be desirable to include in Supplementary tables.

Please see our response to point #1. These identifiers may be found in Supplemental Data 9.

8. In the same solar tracking experiment, in ZT12-16, there seems to be enrichment of ribosomal proteins and translation. A comment on the potential relevance of these findings in terms of novel mechanisms to explore in terms translation links to the joint mechanisms for differential growth by the circadian clock and light would be desirable to include in discussion.

Thank you for this suggestion. We have now examined the time points in which ribosomal and translation GO terms are enriched in both transcriptome experiments. In the heliotropism experiment, we find enrichment for ribosome and/or translation terms on the east sides of stems at ZT12 and on the west sides of stems at ZT20 (Supplementary Data S9). On both east and west sides, the time of enrichment lags both the time of maximal bending growth (Figure 2B, and Atamian et al (2016) Science, Fig. 2C) and the time when cell wall modification GO terms are first enriched (ZT8 and ZT12 on the east sides of stems, and ZT16 and ZT20 on the west sides of stems). We see similar kinetics in our analysis of the phototropism data. Here, there is significant enrichment of hormone signaling, cell growth, and cell wall modification terms is first seen at time ZT4 and ZT5 on the shaded sides of stems. We first see enrichment of translation-related terms at ZT7 on the shaded sides of stems (several hours after the onset of stem bending (Fig 1B)), and these terms remain enriched until ZT15. Together, these results suggest that upregulation of genes encoding the translation machinery occurs after the initiation of bending and is not a driver of asymmetric growth. We have added a new supplemental figure to highlight the timing of distinct GO enrichment terms during phototropism (Figure S3) and briefly discuss this in the results section (lines 187 – 192).

9. In the transcriptional responses to phototropism and heliotropism section. Fig 2G singles out the S15a-like gene, but it may be more suitable to include as examples of genes that could be related to the differential growth responses.

In Fig 3A-B, we selected the *S15a* homolog both because its expression pattern is representative of genes with a significantly later peak on the east sides of stems and because its annotated function is in ribosome assembly, the most significantly enriched GO term for genes with this expression pattern (Supplemental Data S10).

10. Interestingly the authors report that hundred of genes with different amplitudes of expression on opposite sides of heliotropic stems did not show significantly enriched functional categories, and there was limited overlap between genes expressed within different phases and amplitudes in the field. Can the authors comment on the potential impact on other environmental factors (i.e temperature, light intensity) on these set of genes?. How much variation linked to other environmental factors could be present in this study between CE studies of phototropism and the heliotropic response?

Thank you for this comment. It is most likely that the lack of significant GO enrichment for genes with different amplitudes of expression or phases of expression across the stem is because these transcripts have a range of different peak phases of expression, and we find that different types of GO terms are enriched at different phases (See Supplemental Data S5). Our data suggests that the bulk of differential gene expression across the stem is not driven by side-specific environmental cues; we find that more than 70% of the differential expression occurs at night (ZT16 and ZT20) and less than 30% occurs during the day (ZT0, ZT4, ZT8, and ZT12). We have modified the discussion section (lines 599 – 604) to emphasize this point.

11. It is very interesting the identification of phytochrome responses as part of the heliotropic response and the initiation of solar tracking. Would it be possible to extend on the potential impact of the phytochromes in the response, using available genomic datasets in terms of genes responsiveness to R:FR ratios, R-intensity?, also is the potential impact of phy on these genes affecting the phase or affecting the amplitude?

Unfortunately, we don’t have the data to carry out this analysis. We only observed differential expression of genes implicated in phytochrome signaling during the onset of heliotropism (Fig. 4), not during its maintenance. Our heliotropism transcriptome data were collected from plants grown in the field for several weeks, so don’t contain information about genes differentially expressed during initiation of solar tracking.

12. Can the authors comment in the manuscript on the potential differences brought by the light receptors into the growth responses?. They provide with evidence that the onset and maintenance of the heliotropic response can tolerate light quality. Based on what we know about the photoreceptors and their dynamic control of growth, can the authors extend on the potential avenues and mechanisms behind the role of the light quality receptors and day/night differences in growth?

Our analysis of GO categories significantly enriched during heliotropism (Fig. 2M and Supplemental Data S9) didn’t provide us with any insights into growth regulation by specific photoreceptors. The minimal differential expression observed across stems early in the day make us believe that post-transcriptional regulatory mechanisms may play an important role in solar tracking (discussed in lines 658 – 666). We think it premature to speculate further as to which specific photoreceptors that might be involved in this process.

13. Could it be possible to map across the heliotropic responsive genes, with differences in phases and/or amplitude what is the potential contribution of the phys, UVR8 and phots? (even if for selected examples and relating to data across other plant species).

This is also a good suggestion, but even in Arabidopsis we couldn’t find transcriptome studies examining changes in gene expression in response to specific photoreceptors in true stems (rather than hypocotyls). We have found that sunflower hypocotyls are not able to carry out heliotropism, so don't think that a comparison of gene expression responses in Arabidopsis hypocotyls and sunflower stems is likely to be fruitful.

14. In the discussion, it is clearly stated that the focus of the study is transcription but that other mechanisms may be involved in the heliotropic response. Probably worth including together with post-translational cascades, translation (as Fig S7 shows enrichment for some translation related processes).

Please see our response to point #8. Based on our analysis of gene expression kinetics, we don’t believe that translation is driving growth responses.

General comment. It would be desirable to include annotation in all the Supplementary data tables where gene lists are presented.

Thank you for this suggestion. We now include gene annotations for all supplemental data files with lists of sunflower genes.
